# ERBB2 promoter demethylation and immune cell infiltration promote a poor prognosis for cancer patients

**DOI:** 10.3389/fonc.2022.1012138

**Published:** 2022-09-12

**Authors:** Hongting Wang, Yongxu Jiang, Huanhuan Jin, Cunqin Wang

**Affiliations:** ^1^ School of Pharmacy, Drug Research and Development Center, Wannan Medical College, Wuhu, China; ^2^ Anhui Provincial Engineering Laboratory for Screening and Re-Evaluation of Active Compounds of Herbal Medicines in Southern Anhui, Wuhu, China; ^3^ Anhui Provincial Engineering Research Center for Polysaccharide Drugs, Wuhu, China; ^4^ School of Basic Medical Sciences, Shandong University, Jinan, China

**Keywords:** ERBB2, overall survival, demethylation, immune cell infiltration, signaling pathway

## Abstract

**Background:**

Receptor tyrosine-protein kinase erbB-2 (ERBB2) expression is a critical factor for the prognosis of various cancers. ERBB2 enrichment indicates a poor prognosis in some cancer types but could be a favorable prognostic factor in others.

**Methods:**

We analyzed DNA methylation, mRNA, protein, immune cell infiltration, and related signaling pathways using TIMER2.0, GEPIA2, STRING, and UALCAN portal datasets in tumor tissues of diverse cancer types and their matched normal tissues.

**Results:**

ERBB2 promoter demethylation increases transcript protein amplification and promotes a poor prognosis for cancer patients. ERBB2 gain-of-function procures immune cell infiltration for tumor growth and drives away T regulatory cells, which suppress or downregulate induction and proliferation of effector T cells. The downstream signaling pathways, such as tumor proliferation, ECM-related genes, and degradation of ECM, are involved in ERBB2 gene demethylation and immune activation in cancer progression.

**Conclusion:**

ERBB2 gene demethylation leads to a poor prognosis in cancer patients, which is strongly influenced by the composition and abundance of tumor immune cell infiltration. ERBB2 demethylation could be used in clinical practice to identify immune profiles and direct therapeutic strategies.

## Introduction

The receptor tyrosine-protein kinase erbB-2 (ERBB2) proto-oncogene encodes the Her-2 tyrosine-protein kinase receptor, which often binds to other members of the epidermal growth factor family as dimerization partners (1). Abnormal amplification of the ERBB2 proto-oncogene could result in increased tumorigenesis of cancers (2). HER2 overexpression is associated with the poor survival outcomes of breast cancer patients (3, 4). Given the complexity of amplification of Her2, it is critical to perform a pan-cancer analysis of the ERBB2 gene and estimate its correlation with survival of patients. Some studies have revealed that ERBB2 gene enrichment is a critical factor in the progression of cancers (2, 3, 5). However, there is no solid evidence to support ERBB2 gene enrichment or deficiency in influencing the survival of cancer patients. Additionally, the relationship between ERBB2 gene expression and the immune micro-environment of various tumor types is elusive. In the analysis of 5,605 cases of breast cancer, ERBB2 was altered in 12.5% of all genes, with 10.6% of amplifications, 2.4% of mutation, and 0.7% of the combination of amplification and mutation (6). Apparently, ERBB2 mutations in the primary breast tumors were associated with poor outcomes in the OMICS data (7–9).

Chronic tumor-related inflammation damages blood vessels. The regeneration of blood vessels promotes tumor growth, recruitment of immune cells to the tumor site, and secretion of pro-inflammatory cytokines (10–13). Rational knowledge of cancer pathogenesis is critical for seeking new treatments to boost the survival rates of cancer patients. Tumor-infiltrating immune cells are critical for predicting prognosis and efficacy of chemotherapy in many carcinomas (14). Cellular heterogeneity of diverse immune and stromal cell phenotypes contributes to the tumor micro-environment, which is challenged in the dynamic analysis of gene mutation and immune cell interactions (15).

The public Tumor Immune Estimation Resource supplies a set of different tumors to comprehensively evaluate the molecular characteristics of tumor-immune cell interactions in pan-cancer analysis (16–18). The TIMER2.0 revealed the expression of ERBB2 and immune cell infiltration in specific cancers and their correlation with prognosis in different types of cancers. Our results revealed the critical roles of ERBB2 gene demethylation and tumor immune cell infiltration, indicating that the survival of cancer patients could be modulated by improving immune cell infiltration and ERBB2 demethylation in various cancers.

## Methods

### Data collection and preprocessing

The tumor immune estimation resource, version 2 (TIMER2) provides a user-friendly web interface for dynamic analysis and visualization of gene associations, which is of broad utility to cancer researchers (14). Using samples from TIMER2 datasets, it showed the expression of ERBB2 between primary tumor and adjacent normal tissues for cancer subtypes of the TCGA project. The “Expression analysis-Box Plots” module of the Gene Expression Profiling Interactive Analysis, version 2 (GEPIA2) web server showed box plots of the expression between these primary tumors and the corresponding normal tissues from the Genotype-Tissue Expression (GTEx) database (19). Molecular data for 32 TCGA cancer types were downloaded from the TCGA data portal. The UALCAN portal, an interactive web resource for analyzing cancer omics data, allowed us to conduct protein expression analysis from the Clinical proteomic tumor analysis consortium database (CPTAC) (20, 21). Differential genes were selected based on an FDR of ≤0.05 and at least a two-fold difference in expression levels.

### Selection of cancer-specific genes

For the ERBB2 gene of each cancer type, we compared tumor samples with normal samples collectively. The tumor-specific gene set was the union of all cancer types (or subtypes). The Analysis-Box Plots module of the GEPIA2 webserver was used to obtain box plots of the expression between these tumor tissues and the corresponding normal tissues of the GTEx database. Additionally, we used violin plots of the ERBB2 expression in different pathological stages (stage I, stage II, stage III, and stage IV) of PAAD, UCEC, KICH, and KIRC tumors *via* the “Pathological Stage Plot” module of GEPIA2. The log2 [TPM (Transcripts per million) +1] transformed data were applied for the box or violin plots.

### Survival prognosis analysis

The “Survival Map” module of GEPIA2 collected the overall survival and disease-free survival maps of all TCGA cancer types. The median score was used as a cutoff value to separate the high-expression and low-expression cohorts. The “Comparison” module set up the data about the overall and disease-free survival with ERBB2 genetic alteration for the TCGA cancer cases. Kaplan–Meier plots with a log-rank *P*-value were generated.

### DNA methylation analysis

The UALCAN portal was used for analyzing ERBB2 DNA methylation and the pathogenesis of different tumors in the KIRC and UCEC datasets. In the TCGA cases, we observed a significant negative correlation between ERBB2 DNA methylation and gene mutation at the promoter region. Integrating gene expression and DNA methylation data obtained from gene mutation analysis were compared with DNA methylation analysis to identify genes that were significantly altered in DNA methylation (22).

### mRNA and protein analysis

The “Expression Analysis-Box Plots” module of the GEPIA2 webserver was used to obtain box plots of the mRNA and protein expression associated with the ERBB2 gene-phenotype of pan-cancer samples in the GTEx database (23–25). Using the CPTAC database, two types of tumors (KIRC and UCEC) were analyzed. The ERBB2 protein expression was summarized for the significant differences between the normal and cancer groups.

### Immune cell infiltration analysis

The “Immune-Gene” module of the TIMER2 webserver was used to explore the association between ERBB2 expression and immune cell infiltration across all tumors in the TCGA dataset (14). The immune cells and cancer-associated fibroblasts were selected. The EPIC, TIDE, MCPCOUNTER, and XCELL algorithms were applied to assess immune cell infiltration. Additional analysis of KICH and LGG cancers revealed that the tumor immunity is driven by macrophages, mast cells, NK cells, helper T cells, and regulatory T cells in the tumor micro-environment. The P-values and partial correlation values were obtained *via* the purity-adjusted Spearman’s rank correlation test (25).

### Gene and signaling pathway analysis

The correlation between the ERBB2 gene and pathway score was analyzed with Spearman and clinical bioinformatics (www.aclbi.com). The density curve on the right represents the distribution of pathway immune score; the upper density curve represents the distribution of gene expression. The value on the top represents the correlation *p*-value, correlation coefficient, and the correlation calculation method. All signaling pathway analysis was performed using R version 4.0.3. packages, and a *p*-value <0.05 presented a remarkable signaling pathway change and gene mutation.

## Results

### ERBB2 gene mutation in the prognosis of various cancers

**Figure 1:**
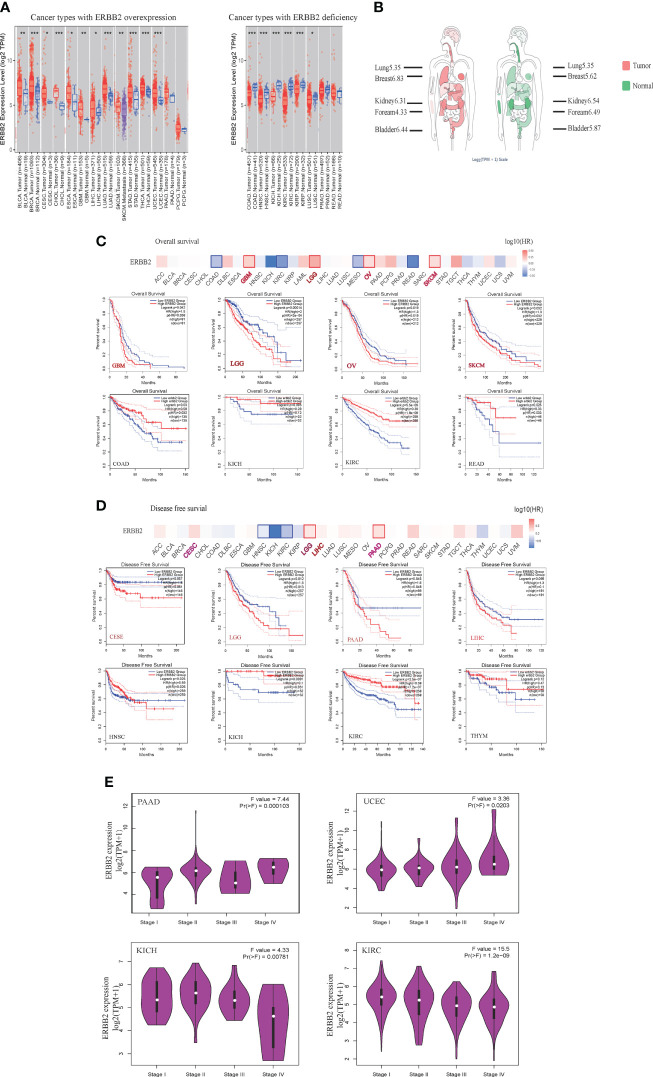
The expression of ERBB2 gene in pan-cancers. **(A)** Distributions of gene expression levels are displayed using box plots. The statistical value analyzed using the Wilcoxon test is annotated by the number of stars (*p-value < 0.05; **p-value <0.01; ***p-value <0.001). **(B)** The distribution graph of the ERBB2 gene in human tissues. The median expression of ERBB2 in tumor and normal samples shows in the body map. Log2 (TPM+1) index marked in different tissues. A larger value shows higher level of ERBB2 gene expression. Overall survival **(C)** and disease-free survival **(D)** analyses of different tumors of the TCGA database using Kaplan-Meier curves. The survival map and Kaplan-Meier curves with positive results draw with the red or blue line. **(E)** Based on the TCGA database, the expression of the ERBB2 gene is present with pathological stages (stage I, stage II, stage III, and stage IV) of PAAD, UCEC, KICH, and KIRC. Log2 (TPM+1) applied for log-scale.

Based on the TCGA, GTEx, and FANTOM5 datasets, we analyzed the ERBB2 gene level of various cancer types (**Figure 1A**). ERBB2 gene shows gain-of-function in some cancer types, such as bladder carcinoma (BLCA), breast carcinoma (BRCA), cervical squamous cell carcinoma (CESC), cholangiocarcinoma (CHOL), esophageal carcinoma (ESCA), glioblastoma (GBM), liver hepatocellular carcinoma (LIHC), lung adenocarcinoma (LUAD), skin cutaneous melanoma (SKCM), stomach adenocarcinoma (STAD), thyroid cancer (THCA), and uterine corpus endometrial carcinoma (UCEC). Conversely, it showed a loss-of-function in other cancer types, for instance, colon adenocarcinoma (COAD), head-neck squamous cell carcinoma (HNSC), kidney chromophobe (KICH), renal clear cell carcinoma (KIRC), kidney renal papillary cell carcinoma (KIRP), lung squamous cell carcinoma (LUSC), and prostate adenocarcinoma (PRAD). In primary tumor samples, 67% of tumors overexpressed the ERBB2 gene, including breast carcinoma, cholangiocarcinoma, hepatocellular carcinoma, cervical squamous cell carcinoma, lung adenocarcinoma, bladder carcinoma, pancreatic adenocarcinoma, lung squamous cell carcinoma, stomach adenocarcinoma, uterine corpus endometrial carcinoma, thyroid cancer, esophageal carcinoma, and rectum adenocarcinoma (**Figure S1A**). However, 31% of cancers showed ERBB2 deficiency in tumor samples, including prostate adenocarcinoma, kidney chromophobe, head-neck squamous cell carcinoma, renal clear cell carcinoma, colon adenocarcinoma, kidney renal papillary cell carcinoma, and lung squamous cell carcinoma (**Figure S1A**). We defined two cancer types according to ERBB2 expression: 1) primary tumor of patients with over-expression of ERBB2 (ERBB2high) and 2) primary tumor of patients with deficient expression of ERBB2 (ERBB2low), and we investigated the correlation of ERBB2 gene expression with the overall survival and disease-free survival of various primary tumors. ERBB2 gene enrichment was correlated with a poor prognosis of cancer patients in some primary tumors, such as glioblastoma, low-grade glioma, ovarian cancer, and skin cutaneous melanoma (**Figure 1C**). Disease-free survival showed a correlation between ERBB2 enrichment and poor prognosis in CESE, LGG, LIHC, and PAAD cancer cases. Interestingly, the ERBB2 enrichment of COAD, KICH, KIRC, THYM, and READ tumor groups has a favorable prognosis based on the survival graphs and disease-free survival analysis (**Figures 1C, D** **)**. A distribution graph of the ERBB2 gene illustrates that the ERBB2 gene has a high-level of expression in some normal tissues, whereas the ERBB2 gene in other tissues shows a low-level of expression in the healthy population (**Figure 1B**). It indicated that the ERBB2 gene was not a malignant indicator in some cancer types, such as kidney chromophobe, renal clear cell carcinoma, and kidney renal papillary cell carcinoma, which is a mutated hetero-morphism of the kidney tissues. Besides, we also explored the correlation of ERBB2 expression and the pathological stages of PAAD and UCEC, which are cancer types with ERBB2 gene gain-of-function, or KICH and KIRH, which are other cancer types with ERBB2 gene loss-of-function, according to the “Pathological Stage Plot” module of GEPIA2 (**Figure 1E**). ERBB2 gene enrichment has an increasing trend in pathological stages I–IV of PAAD and UCEC, while their performance appears to have a decreasing tendency in pathological stages I–IV of KICH and KIRC (**Figure 1E**). A high ERBB2 level also shows a positive relationship with tumor grade and recurrence of PAAD and UCEC, while the deficiency of the ERBB2 gene shows a negative association with tumor grade and recurrence of KICH and KIRC.

### ERBB2 promoter demethylation indicates a poor prognosis in cancer patients

**Figure 2:**
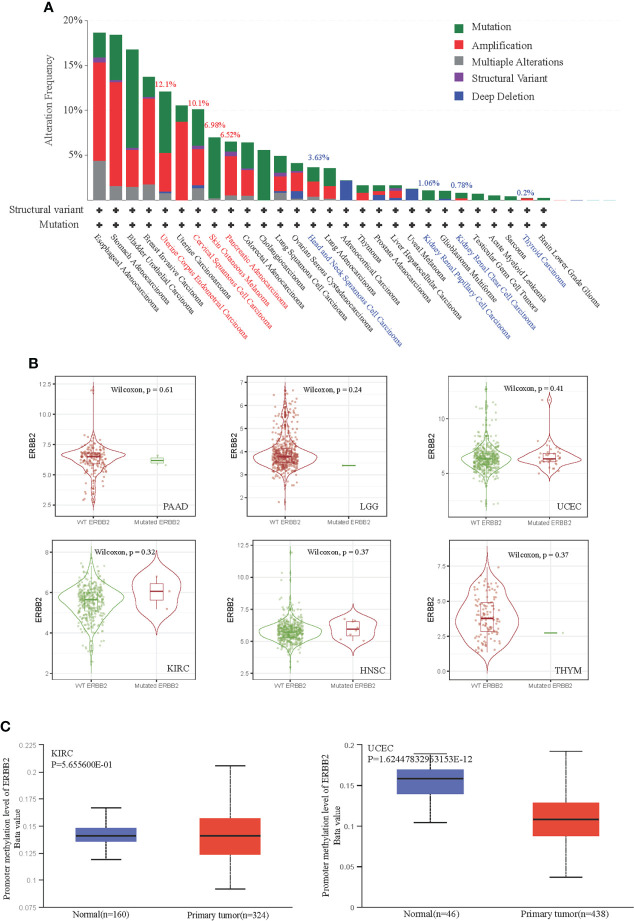
Correlation between ERBB2 gene promoter demethylation and gene mutation in TCGA database. **(A)** ERBB2 alteration frequency of 32 cancer categories is shown based on filtering. It is 12.1%, 10.1%, 6.98%, 6.52% alteration frequency of UCEC, CESE, SKCM, and PAAD; and 3.63%, 1.06%, 0.78%, 0.2% alteration frequency of HNSC, KIRC, KICH, THYM, respectively. **(B)** Mutation cases with biallelic pathogenic alterations of KIRC(n=367), HNSC(n=499), THYM(n=119), PAAD (n=170), LGG(n=511), and UCEC(n=528) are not significant (P>0.05; Wilcoxon rank-sum test) compared with their wild-type cases. **(C)** The level of DNA promoter methylation, ranging from 0 (unmethylated) to 1 (fully methylated). Different deta cut-off value was considered to indicate lower-methylation(Beta value:down to 0.25) or hypo-methylation (Beta-value: 0.3 - 0.25). The normal group of KIRC and UCEC showed lower methylation. However, the primary tumor group of UCEC showed significant demethylation.

The “mutation” type of cancer studies in the pan-cancer cases demonstrate that the gene alteration frequency is about ~5%. To illustrate that ERBB2 gene enrichment leads to a poor prognosis in some cancers but a good prognosis in other cancers, we detailed the gene alteration frequency data for a systematic comparison of ERBB2 gene structural variants across pan-cancers. ERBB2 gene mutation mainly happens in ERBB2^high^ cancers, such as cervical squamous cell carcinoma, stomach adenocarcinoma, breast invasive ductal carcinoma, uterine corpus endometrioid carcinoma, cholangiocarcinoma, and bladder urothelial carcinoma (**Figure 2A**). A high-frequency ERBB2 mutation and amplification result in a poor prognosis for cancer patients, regardless of whether they have more ERBB2 gene copy numbers or not. The percentage of ERBB2 mutation and gene amplification of UCEC, CESE, SKCM, and PAAD is more than that of HNSC, KICH, KIRC, and THYM (**Figure 2A**). Besides, **Figure 2B** revealed that cases with biallelic pathogenic alterations are not significant (P >0.05; Wilcoxon rank-sum test) compared with wild-type cases; they had higher survival in some cancer types and lower survival in other cancer types. We analyzed DNA methylation of two primary tumors, KIRC and UCEC. KIRC is a cancer type with a favorable prognosis, while UCEC is a cancer type with a poor prognosis. Interestingly, the ERBB2 gene of the UCEC presented DNA promoter demethylation, which is a crucial mechanism to trigger the activation of specific genes within tumor progression. Comparatively, there is seldom ERBB2 promoter demethylation in the homologous control (**Figure 2C**). Moreover, ERBB2 promoter demethylation of KIRC did not show a significant change compared to their homologous tissues, indicating a correlation between gene missense activation and promoter demethylation.

### ERBB2 promoter demethylation improves transcript protein amplification

**Figure 3:**
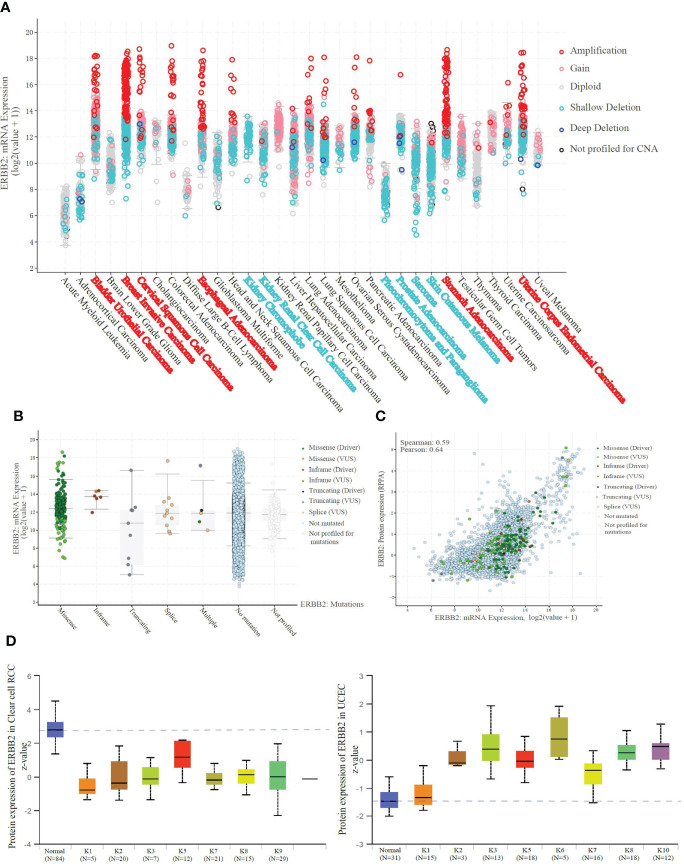
ERBB2 demethylation results in the amplification of mRNA and protein. **(A)** Amplification indicates a high-level amplification (more copies, often focal), and it shows a low-level gain function(a few additional amplification, often broad). Deep deletion indicates a deep loss, possibly a homozygous deletion, and shallow deletion indicates a shallow loss, literally a heterozygous deletion in the pan-cancer analysis. BRCA, STAD, UCEC, BLCA, ESCA, and CESC show specific amplification in the ERBB2 transcription level. And KICH, KIRC, PRAD, SARC, and SKCM present shallow deletion, possibly a heterozygous deletion. **(B)** ERBB2 mRNA expression of 10967 samples from 32 studies was sorted in the available parameters, according to ERBB2 missense, in-frame, truncating, splice, multiple, no mutation, and no profiled. ERBB2 missense is a dominant factor resulting in mRNA amplification. **(C)** Correlation analysis of ERBB2 mRNA expression and protein translation. There is no relationship between ERBB2 mRNA missense or amplification and protein level in the samples. **(D)** Unsupervised clusteringanalysis was performed using the ERBB2 proteins from the CPTAC Confirmatory/Discovery total protein data set across five CPTAC projects. The Mass-spectrometry-based proteomic data to categorize 109 cases into nine different KIRC subtypes. Z-values represent standard deviations from the median score across samples for the given cancer type. ERBB2 protein of K1-K9 subtypes in KIRC displayed a deficiency compared to the normal tissues. Moreover, ERBB2 protein of K1-K10 subtypes in 100 cases of UCEC was performed to be raised compared to the normal tissues, using log-transformed expression values centered on standard deviations from the median score.

We explored the whole transcriptome to identify specific amplification or deletion in the pan caners. The result shows specific ERBB2 mRNA amplification (**Figure 3A**) and truncation (**Figure S1B**) in the ERBB2 transcription level of BRCA, STAD, UCEC, BLCA, ESCA, and CESC. However, more ERBB2 gene deletions (**Figure 3A**) and fewer truncations (**Figure S1B**), possibly a heterozygous deletion, within an ERBB2 transcriptome analysis of KICH, KIRC, PRAD, SARC, and SKCM were observed. ERBB2 mutations promote amino acid changes being encoded at a particular position, resulting in the replacement of one protein building block, especially gene in-frame and gene missense mutations (**Figure 3B)**. Given an evaluation of the regression line, the graph has no slope to reveal the correlation between ERBB2 mRNA transcription and protein translation (**Figure 3C**). In light of mass-spectrometry-based proteomic data analysis from the CPTAC confirmatory cohorts, we compared ERBB2 protein expression between K1–K9 subtypes of KIRC and some kidney tissue cases. All subtypes of KIRC display ERBB2 protein deficiency in 109 samples (**Figure 3D**). Conversely, K1–K10 subtypes of UCEC showed ERBB2 protein overexpression, where their p-values present log-transformed expression values centered on standard deviations from the median, suggesting that the overloading of ERBB2 protein is possible to be a positive link with ERBB2 promoter demethylation.

### The landscape of tumor-infiltrating immune cells with ERBB2 mutation

**Figure 4:**
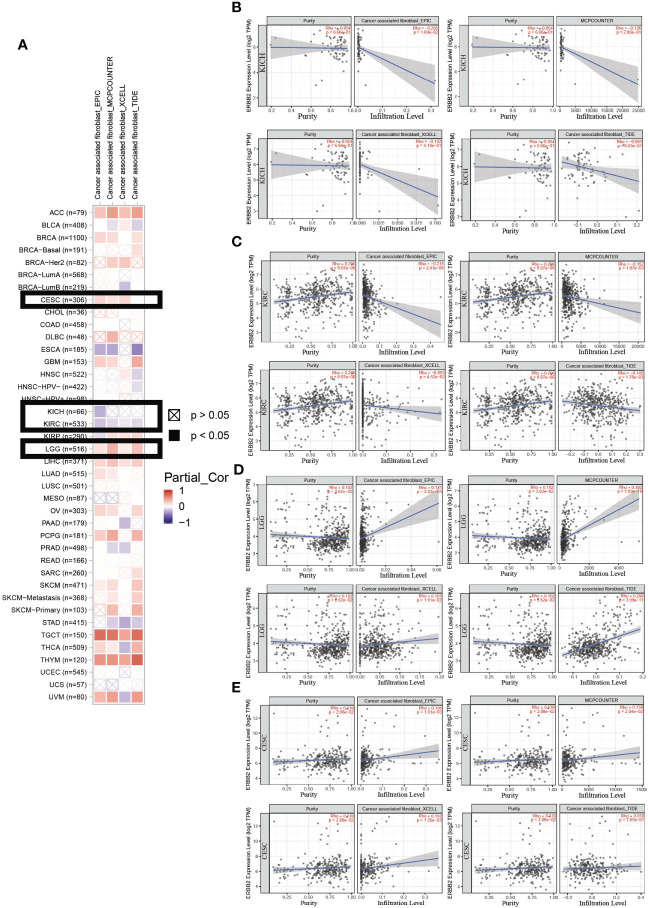
An analysis of the cancer-associated fibroblast infiltration using the four estimation algorithms. **(A)** An example of the scatter plot from the ‘Mutation Module’ displays the difference in TIMER-estimated cancer-associated fibroblast infiltration of KICH, KIRC, LGG, and CESC using the four estimation algorithms, including EPIC, MCP counter, Xcell, and TIDE. Overall, there was a decline in cancer-associated fibroblasts of KIRC **(B)** and KICH **(C)**. Moreover, cancer-associated fibroblasts of LGG **(D)** and CESC **(E)** present an increasing trend in immune cell infiltration.

**Figure 5:**
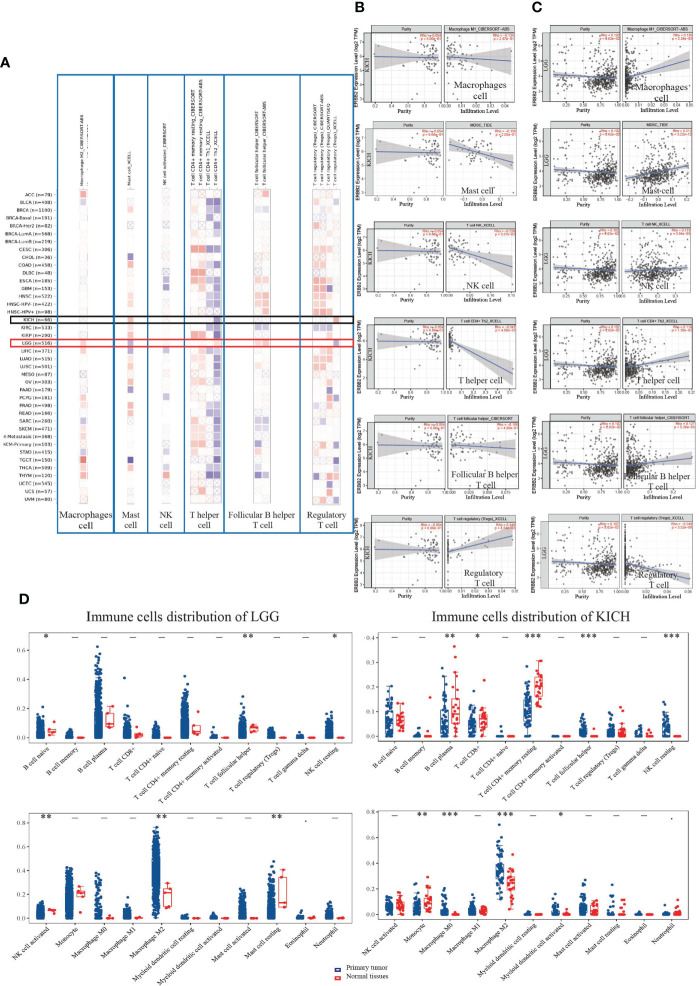
The landscape of immune cell infiltration on differentially expressed ERBB2 in two types of cancers. **(A)** A heat map visualizes immune cell infiltration for assessing different tumors with mutation status of ERBB2 gene, including mast cells, follicular B helper T cells, T follicular helper, T cell regulatory, macrophage cell, and NK cell. **(B)** A scatter plot displays the difference of immune cell infiltration in KICH, such as Tregs, macrophages, NK, mast cells, Th2, and T helper cells. **(C)** A scatter plot displays LGG immune cell infiltration using TIMER-estimate **(D)** The 65 samples of KICH and 513 samples of LGG showed immune cell changes compared to their normal tissues. The abscissa represents immune cell types, and the ordinate represents the expression distribution of immune scores in the two cancer groups. *p<0.05, **p<0.01,***p<0.001, asterisks (*) stand for significance levels. The difference between the two groups presents through the Wilcox test.

We assessed the distribution of immune cells for the correlation between immune infiltrates and the prognosis of cancer patients. In the ERBB2 mutation primary tumors, we detected cancer-associated fibroblast infiltration through the four estimation algorithms, including EPIC, MCP counter, Xcell, and TIDE (**Figure 4A**). Overall, ERBB2 mutation did not activate cancer-associated fibroblast cluster as messengers between the innate and adaptive immune systems. Four estimation algorithms revealed that the more ERBB2 gene enriched in the tumor cluster of KICH and KIRC patients, and less cancer-associated fibroblast infiltrated in the immune cell cluster of KICH and KIRC patients (**Figures 4B, C**). In contrast, the fewer ERBB2 genes expressed in the tumor cluster, the more cancer-associated fibroblasts infiltrated into the immune cell cluster of LGG and CESC (**Figures 4D, E**). Three estimation algorithms detected the distribution of regulatory T cells (Tregs), macrophages (M1), natural killer cells (NKs), mast cells, follicular B helper T cells (Th2), and T helper cells in the two cancer types LGG and KICH (**Figure 5A**). Results revealed that a primary tumor with ERBB2 gene gain-of-function LGG is easier to recruit macrophages, mast cells, NK cells, Th2 cells, and follicular helper T cells, to make them less susceptible to inducers of cell death and to release cytokines that activate other cells (**Figure 5D**). A cancer type with ERBB2 deficiency, KICH presents fewer macrophages, mast cells, NK cells, Th2 cells, and follicular helper T cell cluster infiltration, apparently driving follicular B helper T cell-mediated adaptive immunity (**Figure 5B**). In addition, it comprehensively illustrated that T-cell regulatory clusters have the loss-of-function and decrease-quantity to accompany ERBB2 mutation of LGG patients in **Figure 5C**, which suppress or downregulate induction and proliferation of effector T cells. On the contrary, T-cell regulatory cluster gradually have gain-of-function and increase-quantity in ERBB2 deficient KICH patients, suppressing the immune response to improve the survival rate of KICH patients.

### ERBB2 mutation promotes the activation of downstream signaling pathways

**Figure 6:**
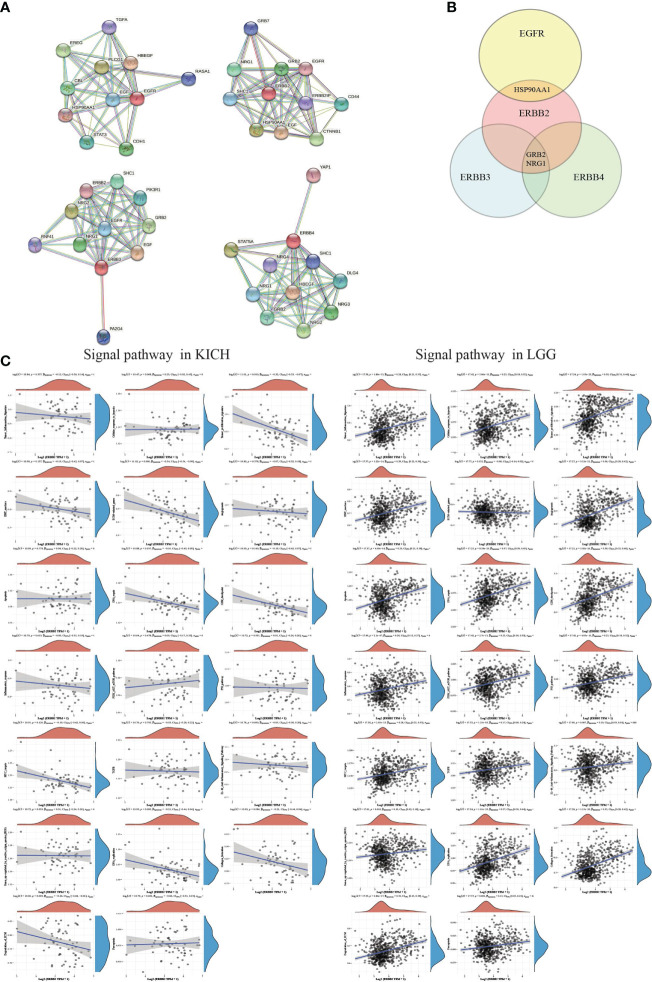
The correlations between individual genes and pathway score. **(A)** The protein-protein interaction network functional enrichment analysis between EGFR family members. **(B)** A Venn diagram identified that the ERBB2 receptor recruits adapter proteins like GRB2 and NRG1, which activates complex downstream signaling cascades using the STRING database. **(C)** A map of KICH and LGG showed the correlation of ERBB2 enrichment and various signaling pathways. The abscissa represents the distribution of the ERBB2 gene expression, and the ordinate represents the distribution of the pathway score. The density curve on the right represents the trend in the distribution of pathway scores. The upper-density curve represents the trend in the distribution of gene expression. The value on the top represents the correlation p-value, correlation coefficient, and correlation calculation method.

ERBB2 binding protein triggers receptor homo- and hetero-dimerization and autophosphorylation on key cytoplasmic residues. The phosphorylated receptor recruits adapter proteins like GRB2 and NRG1, which activate complex downstream signaling cascades (**Figures 6A, B**). The Spearman analysis showed that the crosstalk of the ERBB2 mutation among KICH and LGG primary tumors significantly correlated with some signaling pathways and played an indispensable role in distinct cancer cluster patterns (**Figure 6C**). Three *P-*values of the KICH group revealed that tumor proliferation, the extracellular matrix (ECM)-related gene, and degradation of ECM possessed a more prominent advantage in ERBB2 mutant cancer and progression-free survival. However, many signaling pathways of the LGG group present remarkable changes (*P <*0.05). ERBB2 gene mutation could deteriorate the process of cancer, which involves tumor inflammation signature, cellular response to hypoxia, tumor proliferation signature, EMT marker, angiogenesis, apoptosis, DNA repair, G2M checkpoint, inflammatory response, PI3K/AKT/mTOR pathway, p53 pathway, MYC target, TGFB, IL-10 anti-inflammation signature pathway, ROS upregulated genes, DNA replication, collagen formation, degradation of ECM, and ferroptosis pathways.

## Discussion

Cancer development is a complex process involved in more than one single factor. Abnormal amplification of the ERBB2 proto-oncogene has been condemned as a critical factor resulting in the tumorigenesis of various types of cancers and is related to poor survival outcomes. In a pan-cancer analysis, 67% of primary tumors have a high ERBB2 gene copy number, and 31% of primary tumors present a low level of ERBB2 compared with their non-tumor tissues in the TCGA database. We demonstrated that over-expression of ERBB2 was associated with a poor prognosis for cancers. The ERBB2 copy number increases in the primary tumors of CESE, LGG, LIHC, and PAAD. Interestingly, other cancer patients who suffered from COAD, KICH, KIRC, THYM, and READ also presented more amplification of the ERBB2 gene, had a favorable prognosis of overall survival and disease-free survival.

In the present research, we analyzed the distribution of the ERBB2 gene in various types of tumor populations and comprehensively explored the connections between ERBB2 copy number and clinicopathological characteristics. We demonstrated that the ERBB2 gene mutation is associated with a poor prognosis. We applied several computational methodologies to quantify the infiltration of the immune cells, and specifically identified the downstream signaling pathways associated with immune activation that are prominently elevated in biological pathways. The results showed that a number of normal human tissues have a wide distribution of the ERBB2 gene. Compared to normal kidney tissues, primary kidney tumors do not have a remarkable ERBB2 gene increase in a profiling gene interactive expression. Another analysis also confirmed that two primary tumors, KICH and KIRC, did not present an ERBB2 gene copy number increase. Conversely, these two types of cancers showed a remarkable ERBB2 deficiency, suggesting that ERBB2 overload is not a crucial cause of some cancers, even though 67% of pan-cancers have presented a high ERBB2 gene level. To assess the therapeutic benefit of patients with a ERBB2 mutation, we also compared the ERBB2 gene difference in the various pathological stages of cancer. It was shown that the survival of cancer patients was associated with pathological stages and ERBB2 gene overexpression. In light of the clinical estimation of structural gene variation, we detected that higher ERBB2 mutations and more amplification are frequently associated with a worse prognosis of cancer, regardless of whether these cancers have more ERBB2 gene copy numbers or not. There was a correlation between gene missense and ERBB2 promoter demethylation, which resulted in ERBB2 gene amplification. To identify the correlation between immune infiltrates and prognosis of cancer patients, we demonstrated that ERBB2 gain-of-function of primary tumor boosts macrophages, mast cells, NK cells, Th2 cells, and follicular helper T-cell cluster infiltration, apparently recruits follicular B helper T-cell-mediated adaptive immunity, and gradually drives away T regulatory cells that suppress or downregulate induction and proliferation of effector T cells. The immune cell infiltration could deteriorate the tumor micro-environment and decrease the survival of patients, indirectly suggesting the essential role of the ERBB2 gene demethylation in mediating immune response.

Moreover, we analyzed the downstream signaling pathway involved in ERBB2 gene demethylation and immune activation by assessing the STRING database and comprehensively explored the connections between macrophage-infiltrating immune cells and clinicopathological characteristics, survival outcome, and immunotherapeutic efficacy in cancer therapies, such as tumor proliferation, ECM-related genes, and degradation of ECM. The extracellular matrix is a cell surface-associated three-dimensional macromolecular network that helps cells attach to, and communicate with, nearby cells and plays a crucial role in cell growth, cell movement, and other cell functions. The network provides a dynamic microenvironment surrounding the cell, enabling it to carry on its function and involves a complex series of steps in which cancer cells leave the primary tumor site, resulting in tumor metastasis and a poor prognosis for cancer patients. It was revealed that these signaling pathways possessed more prominent advantages in ERBB2 mutant cancer and progression-free survival. In light of these results, we demonstrated that ERBB2 demethylation could be used in clinical practice to identify immune profiles and direct therapeutic strategies. Nevertheless, more prospective datasets of cancer samples were required to verify our results.

## Conclusions

In summary, we comprehensively analyzed ERBB2 gene mutations using the TCGA database concerning gene-phenotype, gene transcription, protein translation, and tumor-infiltrating immune cells. We systematically established contact between signaling pathways with prognosis and immunotherapeutic efficacy. This integrated analysis demonstrated that patients with ERBB2 overexpressed primary tumors have a poor prognosis and a low survival rate. ERBB2 gene promoter demethylation activated an immunity mechanism and enhanced protein translation in a non-canonical manner, which is a critical factor in the poor prognosis of cancer patients. This study provided novel insights that link immune cell infiltration and ERBB2 promoter demethylation-related signatures to patient survival.

## Data availability statement

The datasets presented in this study can be found in online repositories. The names of the repository/repositories and accession number(s) can be found in the article/**Supplementary Material**.

## Ethics statement

Ethical review and approval was not required for the study on human participants in accordance with the local legislation and institutional requirements. Written informed consent for participation was not required for this study in accordance with the national legislation and the institutional requirements.

## Author contributions

HW conceived the project, implemented and improved the method, and ran all the analyses. HJ and YJ performed a method comparison analysis. CW made vital suggestions to improve the technique and the study’s overall design. All authors discussed the results. HW contributed to the writing of the manuscript. All authors contributed to the article and approved the submitted version.

## Funding

This work was supported by the Key Research and Development Projects of Anhui Province grant (202004a07020042), the Nature Science Research Project of Anhui province grant (KJ2020A0621, YJ20210556), the Project of Anhui administration of traditional Chinese Medicine (2020zcyb10), and the National Natural Science Foundation of China grant (81402818).

## Conflict of interest

The authors declare that the research was conducted in the absence of any commercial or financial relationships that could be construed as a potential conflict of interest.

## Publisher’s note

All claims expressed in this article are solely those of the authors and do not necessarily represent those of their affiliated organizations, or those of the publisher, the editors and the reviewers. Any product that may be evaluated in this article, or claim that may be made by its manufacturer, is not guaranteed or endorsed by the publisher.

## Abbreviations

BLCA, bladder carcinoma; BRCA, breast carcinoma; COAD, colon adenocarcinoma; CESC, cervical squamous cell carcinoma; CHOL, cholangiocarcinoma; CPTAC, clinical proteomic tumor analysis consortium database; ECM, extracellular matrix; ESCA, esophageal carcinoma; ESCC, esophageal squamous cell carcinoma; FTO, fat mass and obesity-associated protein; GBM, glioblastoma; GEPIA2, Gene Expression Profiling Interactive Analysis, version 2; GTEx, Genotype-Tissue Expression; HNSC, head–neck squamous cell carcinoma; KICH, kidney chromophobe; KIRC, renal clear cell carcinoma; KIRP, kidney renal papillary cell carcinoma; LUSC, lung squamous cell carcinoma; LIHC, liver hepatocellular carcinoma; LUAD, lung adenocarcinoma; PRAD, prostate adenocarcinoma; SKCM, skin cutaneous melanoma; STAD, stomach adenocarcinoma; THCA, thyroid cancer; TIMER2, Tumor immune estimation resource, version 2; TPM, Transcripts per million; UCEC, uterine corpus endometrial carcinoma.

## References

[1] Robichaux JP Elamin YY Vijayan R Nilsson MB Hu L He J. Pan-cancer landscape and analysis of ERBB2 mutations identifies poziotinib as a clinically active inhibitor and enhancer of T-DM1 activity. Cancer Cell (2019) 36:444–57.e7. doi: 10.1016/j.ccell.2019.09.001 31588020 PMC6944069

[2] Priestley P Baber J Lolkema MP Steeghs N de Bruijn E Shale C. Pan-cancer whole-genome analyses of metastatic solid tumours. Nature (2019) 575:210–6. doi: 10.1038/s41586-019-1689-y PMC6872491 31645765

[3] Li Z Chen S Feng W Luo Y Lai H Li Q. A pan-cancer analysis of HER2 index revealed transcriptional pattern for precise selection of HER2-targeted therapy. EBioMedicine (2020) 62:103074. doi: 10.1016/j.ebiom.2020.103074 33161227 PMC7670125

[4] Prat A Pascual T De Angelis C Gutierrez C Llombart-Cussac A Wang T. HER2-enriched subtype and ERBB2 expression in HER2-positive breast cancer treated with dual HER2 blockade. J Natl Cancer Inst (2020) 112:46–54. doi: 10.1093/jnci/djz042 31037288 PMC7850037

[5] Kurozumi S Alsaleem M Monteiro CJ Bhardwaj K Joosten S Fujii T. Targetable ERBB2 mutation status is an independent marker of adverse prognosis in estrogen receptor positive, ERBB2 non-amplified primary lobular breast carcinoma: a retrospective in silico analysis of public datasets. Breast Cancer Res (2020) 22:85. doi: 10.1186/s13058-020-01324-4 32782013 PMC7422515

[6] Ross JS Gay LM Wang K Ali SM Chumsri S Elvin JA. Nonamplification ERBB2 genomic alterations in 5605 cases of recurrent and metastatic breast cancer: An emerging opportunity for anti-HER2 targeted therapies. Cancer (2016) 122:2654–62. doi: 10.1002/cncr.30102 27284958

[7] Lawrence MS Sougnez C Lichtenstein L Cibulskis K Lander E Gabriel SB. Comprehensive genomic characterization of head and neck squamous cell carcinomas. Nature (2015) 517:576–82. doi: 10.1038/nature14129 PMC4311405 25631445

[8] Hayes DN Van Waes C Seiwert TY. Genetic landscape of human papillomavirus-associated head and neck cancer and comparison to tobacco-related tumors. J Clin Oncol (2015) 33:3227–34. doi: 10.1200/JCO.2015.62.1086 PMC4586167 26351353

[9] Rusan M Li YY Hammerman PS. Genomic landscape of human papillomavirus-associated cancers. Clin Cancer Res (2015) 21:2009–19. doi: 10.1158/1078-0432.CCR-14-1101 PMC4417456 25779941

[10] Shalapour S Karin M. Immunity, inflammation, and cancer: an eternal fight between good and evil. J Clin Invest. (2015) 125:3347–55. doi: 10.1172/JCI80007 PMC4588298 26325032

[11] Setrerrahmane S Xu H. Tumor-related interleukins: old validated targets for new anti-cancer drug development. Mol Cancer (2017) 16:153. doi: 10.1186/s12943-017-0721-9 28927416 PMC5606116

[12] Siret C Collignon A Silvy F Robert S Cheyrol T André P. Deciphering the crosstalk between myeloid-derived suppressor cells and regulatory T cells in pancreatic ductal adenocarcinoma. Front Immunol (2019) 10:3070. doi: 10.3389/fimmu.2019.03070 32038621 PMC6987391

[13] Haist M Stege H Grabbe S Bros M. The functional crosstalk between myeloid-derived suppressor cells and regulatory T cells within the immunosuppressive tumor microenvironment. Cancers (Basel) (2021) 13:210–44. doi: 10.3390/cancers13020210 PMC7827203 33430105

[14] Li T Fu J Zeng Z Cohen D Li J Chen Q. TIMER2.0 for analysis of tumor-infiltrating immune cells. Nucleic Acids Res (2020) 48:W509–509W514. doi: 10.1093/nar/gkaa407 32442275 PMC7319575

[15] Azizi E Carr AJ Plitas G Cornish AE Konopacki C Prabhakaran S. Single-cell map of diverse immune phenotypes in the breast tumor microenvironment. Cell (2018) 174:1293–308.e36. doi: 10.1016/j.cell.2018.05.060 29961579 PMC6348010

[16] Attalla K DiNatale RG Rappold PM Fong CJ Sanchez-Vega F Silagy AW. Prevalence and landscape of actionable genomic alterations in renal cell carcinoma. Clin Cancer Res (2021) 20:5595–06. doi: 10.1158/1078-0432.CCR-20-4058 PMC8530915 34261695

[17] Sun M Wang Y Zheng C Wei Y Hou J Zhang P. Systematic functional interrogation of human pseudogenes using CRISPRi. Genome Biol (2021) 22:240. doi: 10.1186/s13059-021-02464-2 34425866 PMC8381491

[18] Yu Y Werdyani S Carey M Parfrey P Yilmaz YE Savas S. A comprehensive analysis of SNPs and CNVs identifies novel markers associated with disease outcomes in colorectal cancer. Mol Oncol (2021) 12:3329–47. doi: 10.1002/1878-0261.13067 PMC8637572 34309201

[19] Webb-Robertson BM Bramer LM Jensen JL Kobold MA Stratton KG White AM. P-MartCancer-Interactive online software to enable analysis of shotgun cancer proteomic datasets. Cancer Res (2017) 77:e47–47.e50. doi: 10.1158/0008-5472.CAN-17-0335 29092938 PMC5679244

[20] Cao L Huang C Cui Zhou D Hu Y Lih TM Savage SR. Proteogenomic characterization of pancreatic ductal adenocarcinoma. Cell (2021) 184:5031–52.e26. doi: 10.1016/j.cell.2021.08.023 34534465 PMC8654574

[21] Satpathy S Krug K Jean Beltran PM Savage SR Petralia F Kumar-Sinha C. A proteogenomic portrait of lung squamous cell carcinoma. Cell (2021) 184:4348–71.e40. doi: 10.1016/j.cell.2021.07.016 34358469 PMC8475722

[22] Zhao F Ge F Xie M Li Z Zang C Kong L. FTO mediated ERBB2 demethylation promotes tumor progression in esophageal squamous cell carcinoma cells. Clin Exp Metastasis (2022) 4:623–39. doi: 10.1007/s10585-022-10169-4 PMC9338917 35524932

[23] Peng X Xu X Wang Y Hawke DH Yu S Han L. A-to-I RNA editing contributes to proteomic diversity in cancer. Cancer Cell (2018) 33:817–28.e7. doi: 10.1016/j.ccell.2018.03.026 29706454 PMC5953833

[24] Wei L Jin Z Yang S Xu Y Zhu Y Ji Y. TCGA-assembler 2: software pipeline for retrieval and processing of TCGA/CPTAC data. Bioinformatics (2018) 34:1615–7. doi: 10.1093/bioinformatics/btx812 PMC5925773 29272348

[25] Clark DJ Dhanasekaran SM Petralia F Pan J Song X Hu Y. Integrated proteogenomic characterization of clear cell renal cell carcinoma. Cell (2019) 179:964–83.e31. doi: 10.1016/j.cell.2019.10.007 31675502 PMC7331093

