# Supplementary material for: ERBB2 promoter demethylation and immune cell infiltration promote a poor prognosis for cancer patients
Source: Front Oncol. 2022 Sep 12;12:1012138. doi: 10.3389/fonc.2022.1012138 (PMC9511046; doi:10.3389/fonc.2022.1012138)
Supplement: Supplementary file 1 [file Image_1.pdf]

A

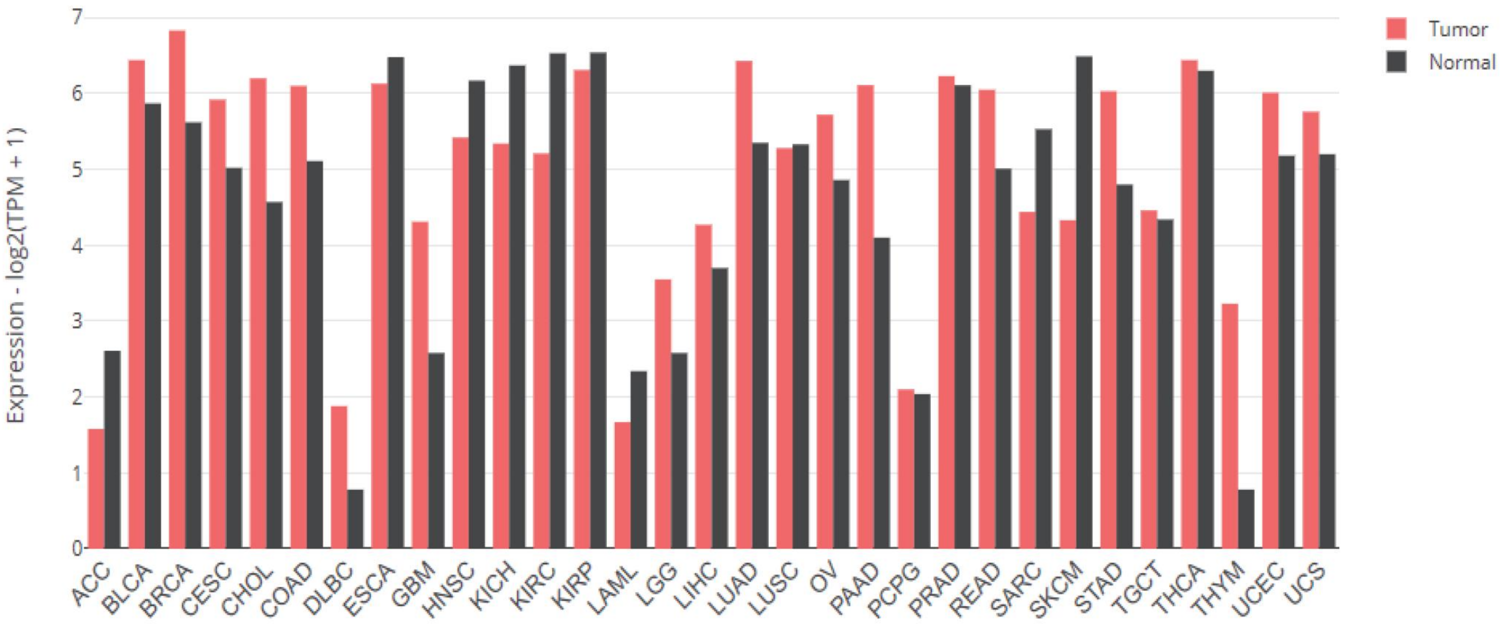


B

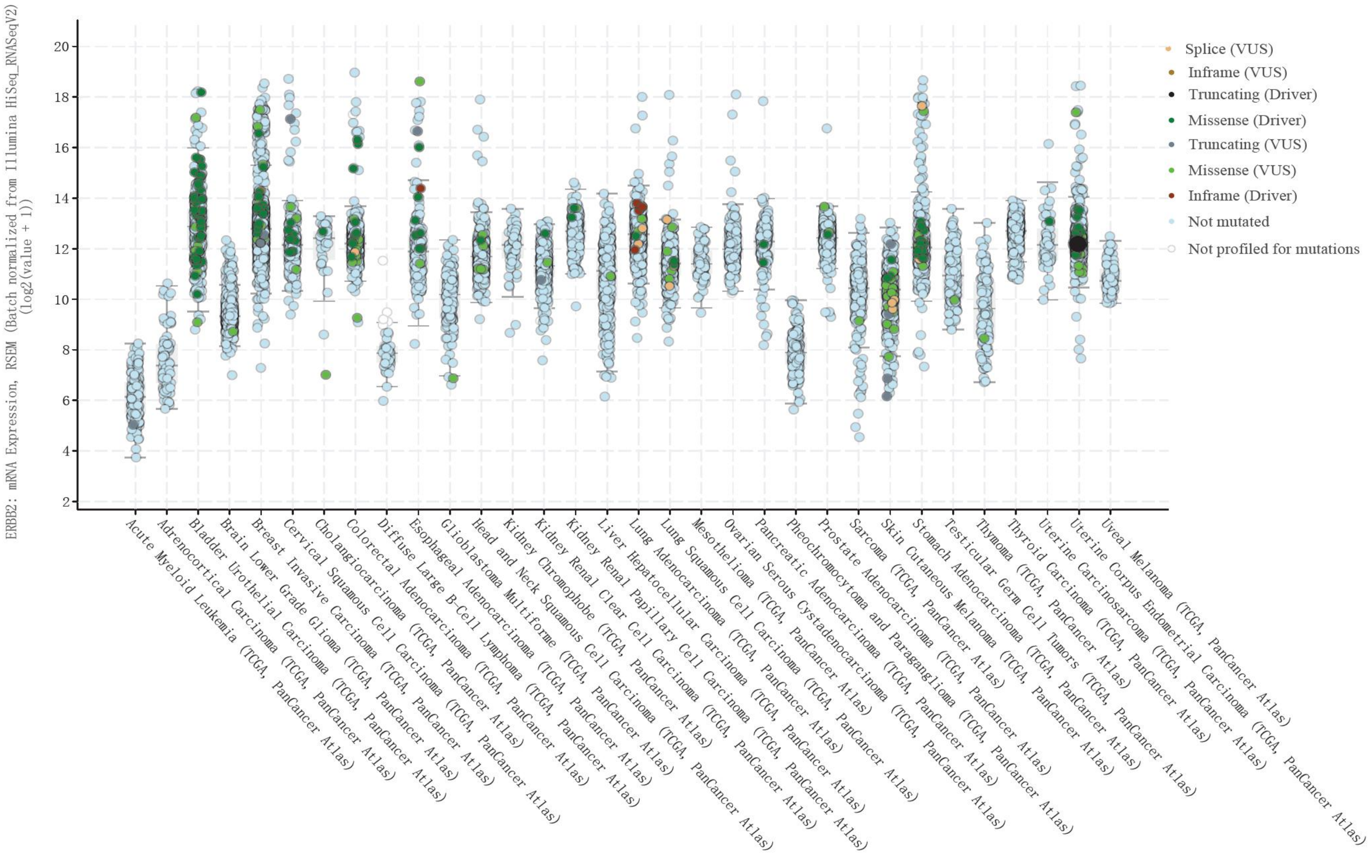


Supplementary figure1

(A)Compare to primary tumor-normal samples, ERBB2 gene expression was performed in pan-cancer analysis.

(B)Whole transcriptome analysis of the ERBB2 for identifing specific truncating or missense actions in pan caners.
